# Unveiling the neuroplastic capacity of the bilingual brain: insights from healthy and pathological individuals

**DOI:** 10.1007/s00429-024-02846-9

**Published:** 2024-09-18

**Authors:** Ileana Quiñones, Sandra Gisbert-Muñoz, Lucía Amoruso, Lucia Manso-Ortega, Usue Mori, Garazi Bermudez, Santiago Gil Robles, Iñigo Pomposo, Manuel Carreiras

**Affiliations:** 1 Biogipuzkoa Health Research Institute, Basque Foundation for Science, San Sebastian, 20009 Spain; 2 Ikerbasque, Basque Foundation for Science, Bilbao, 48009 Spain; 3 ESIC Business and Marketing School, Valencia, Spain; 4 University of the Basque Country, UPV/EHU, Bilbao, 48940 Spain; 5 BCBL, Basque Center on Cognition, Brain, and Language, San Sebastian, 20009 Spain; 6 Biobizkaia Health Research Institute, Bilbao, 48015 Spain; 7 Department of Neurosurgery, Hospital Cruces, Bilbao, 48903 Spain; 8 Department of Neurosurgery, Hospital Quirón salud, Madrid, 28223 Spain

**Keywords:** Bilingualism, Speech production, Neuroplasticity, Brain tumors, fMRI

## Abstract

Research on the neural imprint of dual-language experience, crucial for understanding how the brain processes dominant and non-dominant languages, remains inconclusive. Conflicting evidence suggests either similarity or distinction in neural processing, with implications for bilingual patients with brain tumors. Preserving dual-language functions after surgery requires considering pre-diagnosis neuroplastic changes. Here, we combine univariate and multivariate fMRI methodologies to test a group of healthy Spanish-Basque bilinguals and a group of bilingual patients with gliomas affecting the language-dominant hemisphere while they overtly produced sentences in either their dominant or non-dominant language. Findings from healthy participants revealed the presence of a shared neural system for both languages, while also identifying regions with distinct language-dependent activation and lateralization patterns. Specifically, while the dominant language engaged a more left-lateralized network, speech production in the non-dominant language relied on the recruitment of a bilateral basal ganglia-thalamo-cortical circuit. Notably, based on language lateralization patterns, we were able to robustly decode (AUC: 0.80 ± 0.18) the language being used. Conversely, bilingual patients exhibited bilateral activation patterns for both languages. For the dominant language, regions such as the cerebellum, thalamus, and caudate acted in concert with the sparsely activated language-specific nodes. In the case of the non-dominant language, the recruitment of the default mode network was notably prominent. These results demonstrate the compensatory engagement of non-language-specific networks in the preservation of bilingual speech production, even in the face of pathological conditions. Overall, our findings underscore the pervasive impact of dual-language experience on brain functional (re)organization, both in health and disease.

## Introduction

Speech production is a remarkable skill that appears effortless; however, it involves a complex set of hierarchically organized cognitive processes (Hickok 2012, 2022; Piai and Zheng 2019). These processes encompass concept retrieval and selection, retrieval of syntactic, morphological, and phonological information, post-lexical planning of articulation, as well as self-monitoring and language control mechanisms (Dell 1986; Hickok 2012; Levelt et al. 1999). Neuroimaging research has identified several key neural structures responsible for these functions in healthy individuals, such as the inferior frontal (IFG), superior temporal (STG), and supramarginal gyri (SMG), which are critical for linguistic functions, as well as the cerebellum, primary motor, and somatosensory cortices, which control the mapping between sensory and motor representations (Hickok 2012). While extensively studied in monolinguals (Hickok 2012, 2022; Zhang et al. 2023), research on the neural architecture underlying speech production in bilinguals remains limited. The present study aims to fill this knowledge gap by investigating the neural networks involved in bilingual speech production, offering potential insights into language recovery in bilingual patients with brain pathologies.

Research investigating first and second language production in healthy bilinguals has yielded inconsistent results. Some studies have shown specificities across languages (Gurunandan et al. 2019, 2020; Quiñones et al. 2021; Rodriguez-Fornells et al. 2002; Sierpowska et al. 2018; Xu et al. 2017) while others have revealed overlapping neural substrates (Consonni et al. 2013; Geng et al. 2022; Hernandez et al. 2001; Willms et al. 2011). To shed light on this contradictory scenario, (Tao et al. 2021) conducted a systematic review to summarize functional and structural neuroplasticity findings associated with bilingualism. They concluded that the experience of bilingualism has an impact on brain activity in domain-general control regions (see also Bruin et al. 2021; Pliatsikas and Luk 2016). These areas include the right caudate nucleus, the anterior cingulate cortex, the left parietal lobe, and the bilateral cerebellum. However, some fMRI studies have also revealed functional changes in language-specific regions, including the left STG, left SMG, fusiform gyrus, and IFG (Xu et al. 2017). While this evidence suggests a shared neural substrate albeit with certain specificities for each language, uncertainty persists regarding whether a dual-language experience alters general, non-language-specific mechanisms, as proposed by Tao’s et al. (2021) meta-analysis, or if it also shapes language-specific neural architecture.

Understanding the bilingual brain becomes even more pertinent as the number of bilingual individuals at risk of neurological diseases rises. Brain damage to the language network, such as in the case of brain tumors, can have a significant impact on an individual’s quality of life, affecting one or both languages they speak. The statistics on this matter are alarming: according to the World Health Organization, central nervous system tumors affect around 300,000 individuals per year, many of whom have linguistic profiles that involve multiple languages (Patel et al. 2019). Taking this into account, healthcare centers have incorporated preoperative language mapping protocols into their clinical routines, using functional magnetic resonance imaging (fMRI) along with simple production tasks (De Witte et al. 2015; Shapiro et al. 2005). This non-invasive technique provides valuable information on the configuration and functional lateralization of the language network, enabling neurosurgeons to design personalized interventions to minimize the risk of postsurgical neurological sequelae while maximizing tumor resection (Stopa et al. 2020).

However, there is currently no established and validated protocol for preoperative language mapping in the bilingual brain. Most studies on preoperative mapping in brain tumor patients have been conducted in the official language of the country where the patient was treated, regardless of the patient’s dominant language or the different languages they speak (Fernandez-Coello et al. 2021; Pascual et al. 2023; ReFaey et al. 2020). To address this gap, we developed and validated a picture-naming task called MULTIMAP, which consists of an open-source database of pictures representing objects and actions, normed for ten different languages while controlling for linguistic measures such as name agreement, frequency, length, and substitution neighbors. This task minimizes the linguistic distance between language pairs allowing multilingual pre- and intraoperative mapping in brain tumor patients (Gisbert-Muñoz et al. 2021).

In the present study, we created an fMRI sentence completion task based on the MULTIMAP pictures. This was used to measure brain activity in healthy Spanish-Basque simultaneous bilinguals and bilingual patients with brain tumors while performing the task in either their dominant or non-dominant language. This study pursues two specific objectives. Firstly, we aim to explore whether the two coexistent languages in healthy bilinguals engage similar or different neural networks in terms of regional activation and lateralization patterns. By analyzing this, we can gain valuable insights into the neurobiology of bilingual speech production and provide normative data to better understand potential neuroplastic changes in patients. Secondly, we utilize the same task to investigate how neuroplastic compensatory mechanisms, triggered by brain tumors affecting the language-dominant hemisphere, differentially impact the dominant and non-dominant language in bilingual patients.

Given the contradictions in previous fMRI evidence, we will evaluate two possible hypotheses regarding language production in the group of healthy simultaneous bilinguals. First, if the dominant and non-dominant language rely on a common neural substrate, we should not find differences across languages. Alternatively, considering studies that have shown differences between languages, it is plausible that certain parts of the healthy language network are modulated depending on the language being used. Specifically, we predict a coadjuvant recruitment of domain-general networks involved in executive functions and language control mechanisms in non-dominant speech production task (Abutalebi and Green 2016; Bice et al. 2020; Sulpizio et al. 2020; Tao et al. 2021). This effect could be reflected in the activation pattern of areas such as the basal ganglia, and the cerebellum, which are crucial components of these networks (Burgaleta et al. 2016; Camerino et al. 2022; Murphy et al. 2022; Pliatsikas et al. 2017). Testing these hypotheses will enable us to clarify whether the distinctions between languages are limited to domain-general circuits or if they also impact language-specific areas.

Then, taking the data from the healthy individuals as a baseline, we will investigate how the two languages are distributed in a group of bilingual patients with gliomas affecting critical regions for language processing. Considering previous evidence in clinical populations, we expect to uncover how functional neuroplasticity mediates the engagement of the non-dominant contralateral hemisphere as well as potential ipsilateral changes induced by the lesion (Deverdun et al. 2020; Quiñones et al. 2021; Van Dokkum et al. 2019). These findings will contribute to the discussion on the need for personalized pre- and intra-operative multilingual functional mapping strategies, which are crucial for avoiding language-specific cognitive impairments that may differentially affect the dominant and non-dominant language.

## Materials and methods

### Ethics statement

The study protocol was conducted according to the guidelines of the Declaration of Helsinki and approved by the Ethics Board of the Ethics and Scientific Committee of the Basque Center on Cognition, Brain, and Language, BCBL (protocol code PI2020022, date of approval: May 26, 2020). Informed consent was obtained from all subjects involved in the study.

### Healthy sample

The sample of healthy controls was composed of 20 healthy Spanish–Basque highly proficient bilinguals (12 female), with ages ranging from 19 to 36 years (mean = 25.25, SD = 5.76).

All participants had right-handed dominance, normal or corrected to normal vision, and no history of psychiatric, neurological disease, or learning disabilities. The Basque population is a very special cohort in terms of linguistic profile as all individuals are exposed to Spanish and Basque from a very early stage, regardless of which language they learn first. This scenario offers a compelling opportunity to explore the influence of sociolinguistic richness on brain architecture. The dominance, usage, and exposure to Basque and Spanish are intricately linked to the locality of residence. However, according to the latest sociolinguistic survey conducted by the Basque Government, 51.8% of the population over 16 years of age exhibits Basque dominance with a high proficiency in Spanish, including 28.3% identified as simultaneous bilinguals. Conversely, 17.7% of the population demonstrates Spanish dominance with some knowledge of Basque, primarily in comprehension rather than production, while 30.5% is Spanish dominant without knowledge of Basque

Extensive discussions have focused on how variables such as the age of acquisition and level of proficiency impact the neural circuits underlying bilingual language processing (Bruin et al. 2021; Perani and Abutalebi 2005). Our study boasts several strengths that enhance its validity and reliability. Foremost, we meticulously curated a sample of simultaneous bilingual individuals, adhering to stringent inclusion criteria. These criteria included: (1) acquisition of Spanish and Basque before the age of 3; (2) proficiency levels exceeding a score of 4 in the BEST semi-structured interview for both languages (on a scale of 1 to 5, with 5 indicating maximum proficiency); and (3) similar usage percentages for both languages, (i.e., approximately 40% each). Additionally, we controlled for exposure to and acquisition of other languages. While all participants in our study demonstrated some level of English proficiency (10-point Likert self-rating proficiency scale averaged across listening, speaking, reading, and writing = 6.69 (SD = 1.13), it occupies a less dominant position compared to the other two languages (age of acquisition = 5.35 (SD = 3.07); daily exposure = 11.76% (SD = 8.09).^1^.

Language proficiency was measured using the Basque, English, and Spanish picture naming standardized tests [BEST] (Bruin et al. 2017), and a short semi-structured interview conducted by a multilingual linguist with experience in assessing language proficiency, who rated each participant’s skills from 1 to 5 in both languages. To be included in the sample, participants had to score 4 or 5 on the interview. We also collected data on their daily exposure to both languages and asked them to self-rate their proficiency on a 10-point Likert scale for listening, speaking, reading, and writing in both Spanish and Basque. These ratings were averaged to obtain the self-rating score. Finally, participants completed Spanish and Basque versions of the LexTALE, a short lexical decision test that has been shown to provide good estimates of language proficiency (Izura et al. 2014). Scores per language are reported in Table 1, as well as the behavioral results of MULTIMAP. It should be noted that there were no significant differences between languages for any of the variables. Determining the dominant language in simultaneous bilinguals is inherently complex. However, guided by the prevalent exposure to and use of Spanish over Basque at the time of assessment, we designated Spanish as the primary dominant language for these individuals. Thus, throughout the manuscript, we use the term dominant language to refer to Spanish and non-dominant language to refer to Basque.

**Table 1:** Overview of the language assessment scores of the healthy participants. The self-ratings are on a 10-pointlikertt scale and were averaged across listening, speaking, reading, and writing

Language assessment	Spanish	Basque	T test (*p*)
Age of acquisition	0.30 (0.90)	1.60 (1.74)	-0.76 (0.45)
Daily exposure (%)	49.47 (19.85)	40 (21.08)	1.43 (0.16)
Self-ratings	9.51 (0.60)	9.04 (0.99)	1.74 (0.09)
MULTIMAP (picture naming)	95.35 (4.89)	93.32 (4.99)	2.08 (0.15)
LEXTALE (lexical decision)	93.25 (5.18)	91.26 (6.08)	2.02 (0.35)

### Clinical population

Ten Spanish–Basque bilingual patients with low-grade glioma (LGG) took part in this study. This type of primary nervous system tumor evolves slowly which enhances structural and functional neuroplasticity and causes a delay in the onset of cognitive symptoms. Therefore, LGG patients constitute an ideal in vivo pathological model to investigate neuroplasticity associated with a major architectural alteration of a brain network.

**Fig. 1:**
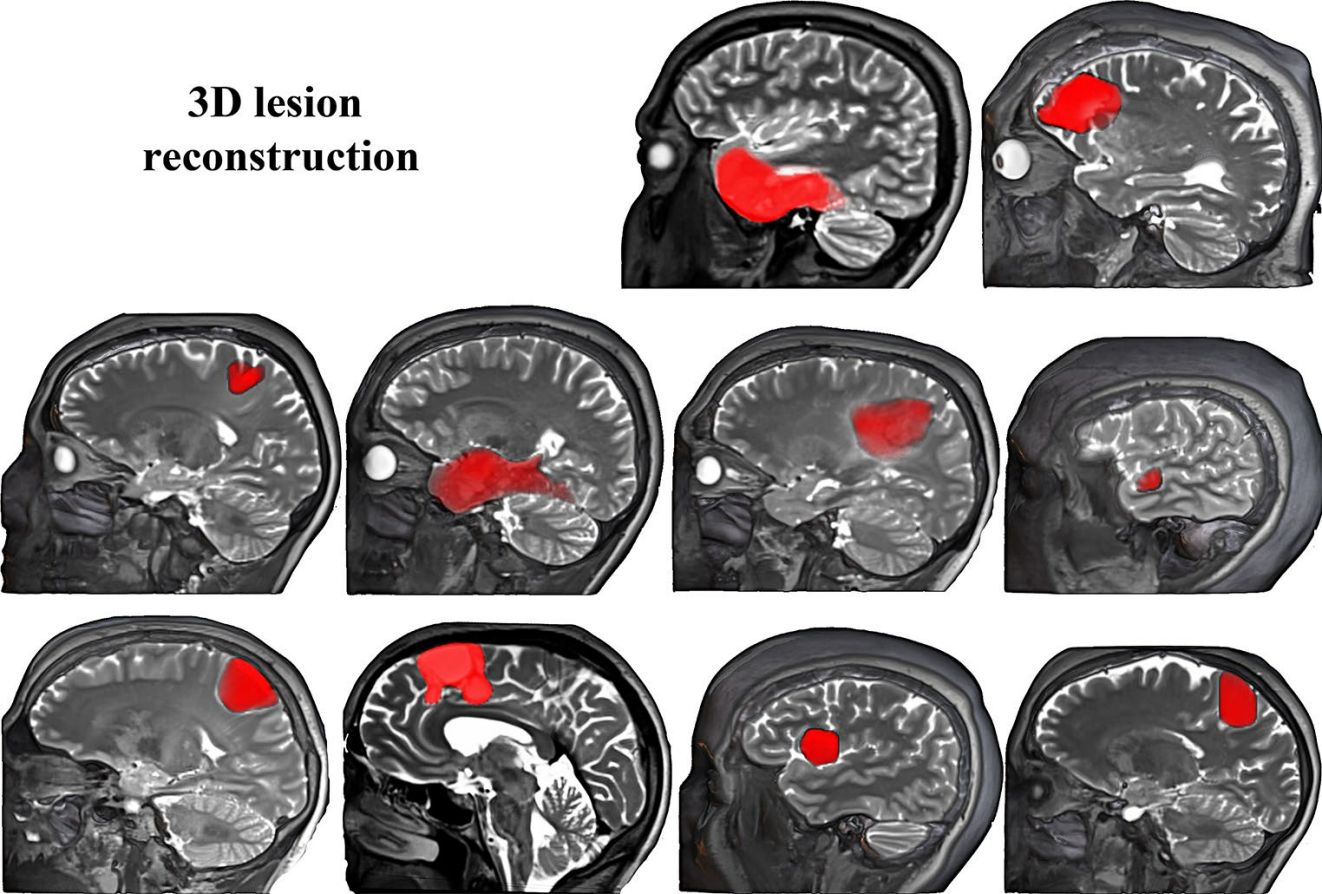
3D lesion reconstruction for the ten bilingual patients

Patients were recruited at the Hospital Universitario Cruces Bilbao (Spain), where they received their diagnosis and underwent awake brain surgery for tumor resection, with intraoperative cortical and subcortical direct stimulation to establish functional boundaries. The initial neurological examinations at the hospital revealed no motor, somatosensory, or linguistic deficits. They all had normal hearing and normal or corrected to normal vision. Individual patients’ demographics and clinical characteristics are summarized in Table 2 (see Fig. 1 for 3D reconstructions of the lesions). Data included in the current research is part of their presurgical examination.

**Table 2:** Individual demographic features

Case	Age (years)	Gender	Studies (years)	Spanish AoA	Basque AoA	Proficiency in % (Spanish)	Proficiency in % (Basque)	Tumor location
P01	38	Female	16	0	3	100	72.31	Frontal
P02	56	Male	10	0	0	98.46	90.77	Frontal
P03	46	Male	14	0	0	100	78.46	Frontal
P04	43	Male	14	0	0	96.92	95.38	Frontal
P05	46	Female	12	0	3	100	98.46	Parietal
P06	32	Female	17	0	3	100	76.92	Parietal
P07	41	Male	16	0	3	100	96.92	Parietal
P08	38	Female	14	0	2	98.46	75.38	Temporal
P09	22	Male	12	0	0	95.38	92.31	Temporal
P10	23	Male	14	0	0	100	95.38	Temporal

#### Bilingual sentence completion test used to characterize the language network

To characterize the language network, we employed a sentence completion task based on MULTIMAP (Gisbert-Muñoz et al. 2021). Specifically, we used a subset of 30 objects and 30 actions, that had been matched between and within our target languages. Target words were matched on frequency, number of orthographic neighbors, and length (i.e., 5–8 characters). In addition, the stimuli were controlled for visual complexity, familiarity, and naming agreement (above 80%). Values for imageability and concreteness were high for both target nouns (mean imageability = 6.21, SD = 0.33; mean concreteness = 5.96, SD = 0.43), and verbs (mean imageability = 5.27, SD = 0.55; mean concreteness = 4.66, SD = 0.64).

The task included four separate blocks (60 items per block): object naming in Spanish and Basque, where the participants saw a picture of an object preceded by the text “Esto es…” or “Hori da…”*—*“This is…” in Spanish or Basque, respectively; and action naming in Spanish and Basque, where the participants saw a picture of a person carrying out an action, introduced by the pronominal phrase “Él…” / “Ella…” or “Hark…”*—*“He…” or “She…” in Spanish or Basque. Participants had to complete the sentences with the noun or verb depicted in the picture. In the case of objects, the noun had to agree in number and/or gender, and for actions, they had to generate a finite verb form in the 3rd person singular.

This type of task has been extensively used to investigate the brain mechanisms underlying language production as well as to assess language-function integrity in pathological populations (Amoruso et al. 2021; Geng et al. 2023; Lubrano et al. 2014; Miceli et al. 2002; Quiñones et al. 2021; Rofes et al. 2017). The stimuli were visually presented in the center of the screen for two seconds, followed by an inter-stimulus interval between two and eight seconds (mean ISI = 5.46 s). Object naming and action naming were tested in different blocks to avoid BOLD changes associated with task switching and differences in the attentional burden across lexical categories (i.e., object naming and verb generation). To minimize the interference effect across languages, the two languages were tested in different sessions. The order in which objects and actions blocks were administered within a session was counterbalanced across participants. We used MATLAB version 2012b and Cogent Toolbox (http://www.vislab.ucl.ac.uk/cogent.php) to present the images (the stimuli, the Matlab script, and its compiled version are available for use at https://git.bcbl.eu/sgisbert/multimap2).

#### Behavioral data analysis

Participants’ responses were recorded to measure accuracy and reaction times (RTs) per trial. An open-source in-house software (“SPONGE”, available at https://github.com/Polina418/Audio_processing) was used to semi-automatically detect speech onsets. RTs were measured as the interval between picture presentation and the onset of the participant’s oral response. Erroneous responses or utterances containing disfluencies were excluded from the final analyses. Paired-sample *t*-tests were run on accuracy and RTs to compare Spanish and Basque production performance.

#### MRI/fMRI data acquisition

All participants –healthy controls and patients– underwent an MRI session in a 3T Siemens Magnetom Prisma Fit scanner (Siemens AG, Erlangen, Germany). High-resolution T1- and T2-weighted images were acquired with a 3D ultrafast gradient echo (MPRAGE) pulse sequence using a 64-channel head coil with the following acquisition parameters for T1: 176 contiguous sagittal slices, voxel resolution 1 × 1 × 1 mm3, Repetition Time (RT) = 2530 ms, Echo Time (ET) = 2.36 ms, Image columns = 256, Image rows = 256, flip angle (Flip) = 7˚; and for T2: 176 contiguous sagittal slices, voxel resolution 1 × 1 × 1 mm3, RT = 3390 ms, ET = 389 ms, Image columns = 204, Image rows = 256, Flip = 120˚. For each participant, the origin of the T1/T2 weighted images was set to the anterior commissure and co-registered using the T1 as reference. To estimate the transformation matrix required for normalizing each individual’s images to MNI space, we combined T1 and T2 images and employed the unified segmentation, bias correction, and spatially normalization approach implemented in SPM. Echo-planar functional images were recorded using the following multiband sequence specifications: number of scans = 203; number of slices = 72; voxel size = 2 mm3 isotropic; ET = 29 ms; repetition time RT = 1.8 s; Field of View (FoV): 192 mm; matrix = 864 × 864; Flip = 73 degrees; acceleration factor = 1; Echo spacing = 10.42 ms. In order to guarantee steady-state tissue magnetization, the first six volumes of each functional run were discarded.

#### GLM-based functional MRI data analysis

*Functional event-related data were analyzed using SPM12 and related toolboxes* (http://www.fil.ion.ucl.ac.uk/spm). Raw functional scans were slice-time corrected taking the middle slice as reference, spatially realigned, unwarped, coregistered with the anatomical T1 and normalized to MNI space using the unified normalization segmentation procedure. Global effects were then removed using a global signal regression analysis (Macey et al. 2004), after which the data were smoothed using an isotropic 8 mm Gaussian kernel. The resulting time series from each voxel were high-pass filtered (128s cut-off period).

Statistical parametric maps were generated using a univariate general linear model with a regressor for each stimulus type obtained by convolving the canonical hemodynamic response function with delta functions at stimulus onsets, also including the six motion-correction parameters as regressors of non-interest. The stimuli onsets included different components per session. The first corresponded to the onset of each sentence trial and was modeled as a single regressor, independently of the experimental conditions. The next corresponded to the different experimental manipulations – i.e., Spanish objects, Spanish verbs, Basque objects, and Basque verbs.

To increase the reliability of the first-level analysis, GLM parameters were estimated using the FAST model which uses a dictionary of covariance components based on exponential covariance functions in the context of the restricted maximum likelihood estimation (Olszowy et al. 2019). Contrast images for each of the conditions compared to the fixation baseline were submitted to the second level Flexible Factorial Design using Language (*Spanish and Basque*) as within-subject factor and subjects as between-subject factor. Each participant was recorded twice for each of the two languages. Thus, for each person we had four samples, giving a total of 80 T-maps that feed the second-level statistical analyses. Each of these maps was estimated from 253 time points. Population-level inferences were tested using a threshold of *p* < 0.001 uncorrected with a voxel extent higher than 50 voxels, then the statistical table was analyzed, and the p and k values were re-calculated to identify clusters with a significant p-value after correction for multiple comparisons using family-wise error rate (FWER, *p* < 0.05) (Nichols and Hayasaka 2003). All local maxima are reported in the results tables as MNI coordinates (Evans et al. 1993).

#### Laterality index estimation based on fMRI data

Language laterality index (LI) is typically calculated globally, by assessing the collective activity across all perisylvian regions that comprise the language network. However, language production is not exclusive to the left hemisphere, and the lateralization pattern depends on the language processing component we are looking at (Bozic et al. 2010; Hickok and Poeppel 2004, 2007; Poeppel 2014a; Turkeltaub and Branch Coslett 2010). Left and right superior temporal areas, for instance, are equally activated in speech perception and lexical-level comprehension tasks (Binder et al. 2000). Thus, it becomes increasingly important to have measures of functional lateralization that characterize each region within the language network, rather than global network lateralization measures. In clinical settings, regional LIs can provide critical information for neurosurgical planning (Brumer et al. 2020) helping to minimize postoperative neurological risk (De Witt Hamer et al. 2012).

To test whether speech production across languages produced similar or different patterns of brain lateralization, we estimated LI per ROIs for the critical contrasts (i.e., Spanish and Basque). Following the threshold-independent bootstrapping approach implemented in the Laterality Index SPM Toolbox (Wilke and Lidzba 2007), for both languages, LI was estimated as LI = (left − right)/(left + right), resulting in positive values for left-dominance and negative values for right-dominance (Bradshaw et al. 2017). This involves iterative resampling and estimation of LIs across multiple threshold levels for all possible right/left sample combinations. To reduce the effect of outliers, trimmed means taken from the middle 50% of the t-value distribution were used as the final LI scores (Wilke and Lidzba 2007). The 54 bilateral ROIs used as spatial constraints were built in MNI space using the AAL atlas covering all cortical and subcortical space. Statistical comparisons were performed keeping patients and healthy controls in separate designs to avoid potential effects due to the differences inherent to the different populations. A repeated measures ANOVA was performed with LI scores for the 54 ROIs as dependent variables. Two within-subject factors were included: (1) Language (Spanish and Basque) and (2) Regions of Interest (54 ROIs). Pairwise comparisons were calculated as a post-hoc analysis applying Bonferroni correction for multiple comparisons.

#### Machine-learning approach

We used a data-driven supervised machine-learning classification approach to test whether the two languages (i.e., Spanish and Basque) could be decoded (“brain-read”) from laterality brain activity signatures. To this end, we used the Extreme Gradient Boosting (XGBoost) algorithm (Chen and Guestrin 2016). This tree-based algorithm creates and combines individually weak yet complementary classifiers, to produce a robust estimator. Of note, it has been recently shown (L. Zhang and Zhan 2017; Zhang et al. 2021) that XGBoost is robust with small and large training sets, outperforming other more well-known classifiers such as Random Forest and Support Vector Machine. Furthermore, this algorithm exhibits high predictive performance for binary classifications using language-related task-based neural activity (Torlay et al. 2017). We fed the machine learning classifier with the LIs obtained for each of the 54 ROIs. First, we standardized brain features using the robust scaler method (i.e., yielding variables with both a zero mean and median, and a standard deviation of 1). Then, to limit biases and obtain more robust results, we employed a *k*-fold validation approach (*k* = 5) using 85% of the sample for training, and 15% hold-out sample as an independent testing set. Importantly, this testing set was never used for hyper-parameter tuning, or feature selection. The data were partitioned using stratified random selection, thus preserving the proportion of labels per condition (Poldrack et al. 2020).

Due to the high dimensionality of the classification problem (i.e. many features and few instances), we employed a feature reduction strategy using the recursive feature elimination method (RFE), aiming to remove redundant non-informative variables and prevent potential overfitting problems (Saeys et al. 2007). Importantly, this feature reduction was applied only to the training set within the cross-validation scheme. Importantly, the resulting sample-to-feature ratio met the recommended N-1 criterion, where N denotes the number of samples used in the training set – namely, 60 samples for training with 8 features (Hua et al. 2005). Additionally, within the cross-validation scheme, we performed an hyper-parameter tuning within the training set using the Grid Search method (Pedregosa et al. 2011).

Classifier performance is reported as mean and SD obtained upon 10 iterations with different random partitions of the data (i.e., using different random seeds). Following state-of-the-art guidelines to report machine-learning results (Uddin et al. 2019), we computed classification accuracy, along with the area under the curve (AUC) of the receiver operating characteristic (ROC) curve and the confusion matrix, capturing the sensitivity and specificity of the classifier. Finally, to enhance the interpretability of the model, we computed feature importance through SHAP (SHapley Additive exPlanations) values (Lundberg and Lee 2017). SHAP values provide an estimate of the impact of each feature on the model’s predictions. All analyses were implemented using the Scikit-learn library (v. 0.22.1) in Python.

## Results

### Characterizing bilingual language production in healthy individuals

#### Behavioral performance

The accuracy showed no differences across languages [*t*_(39)_ = 1.84, *p* = 0.18, *n*^*2*^ = 0.026], indicating comparable performance for Spanish (mean = 95.35%, SD = 5.03) and Basque (mean = 93.32%, SD = 7.48). Similarly, the *t*-test performed on the RTs showed no significant effects between languages [*t*_(39)_ = 0.02, *p* = 0.90, *n*^*2*^ = 0.00003], indicating similar naming latencies for Spanish (mean = 1062.97 ms, SD = 414.79) and Basque (mean = 1049.71 ms, SD = 407.28).

#### Activation-based cross-language effects

**Fig. 2:**
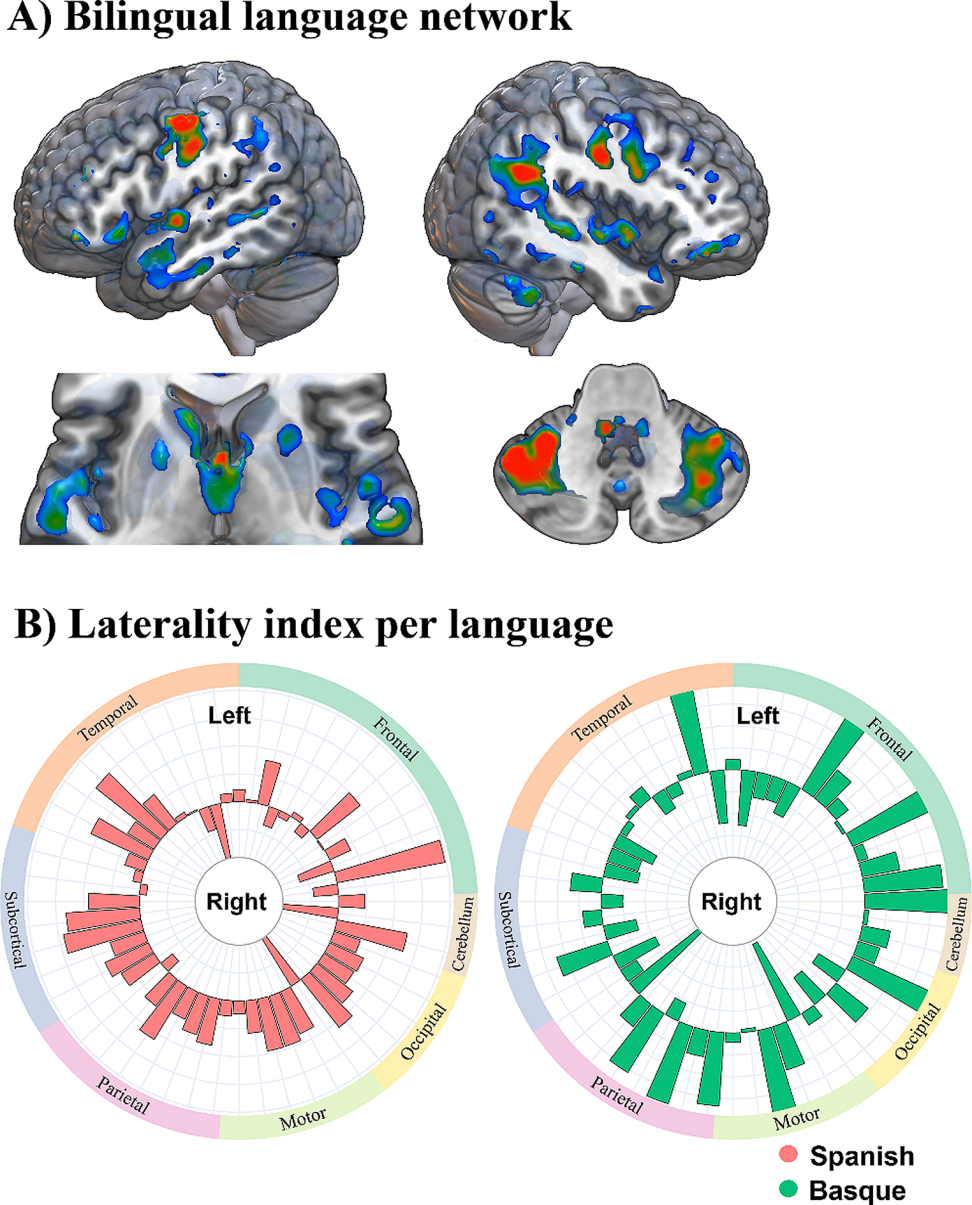
(**A**) Network supporting language production in healthy bilinguals. The statistical parametric map created from the contrast between all conditions versus the null distribution is projected on the MNI single-subject T1 image using the glass brain configuration parameters of MRIcroGL (https://www.nitrc.org/projects/mricrogl). All clusters depicted are statistically significant with a p-value corrected for multiple comparisons (FWER, *p* < 0.05). (**B**) Circular charts showing lateralization indices for Spanish (in red) and Basque (in green) in healthy bilinguals

Language production – as compared to a visual baseline – produced a widespread pattern of activation that included regions in both hemispheres. As expected, this contrast comprised areas such as the pars orbitalis within the IFG, the SMA, the precentral and postcentral gyri, the paracentral lobule, the STG and MTG, the superior parietal gyrus, and the cerebellum. All these areas were bilaterally recruited in both Spanish and Basque (see Fig. 2). However, the activation pattern of these regions was modulated as a function of the language being used.

**Fig. 3:**
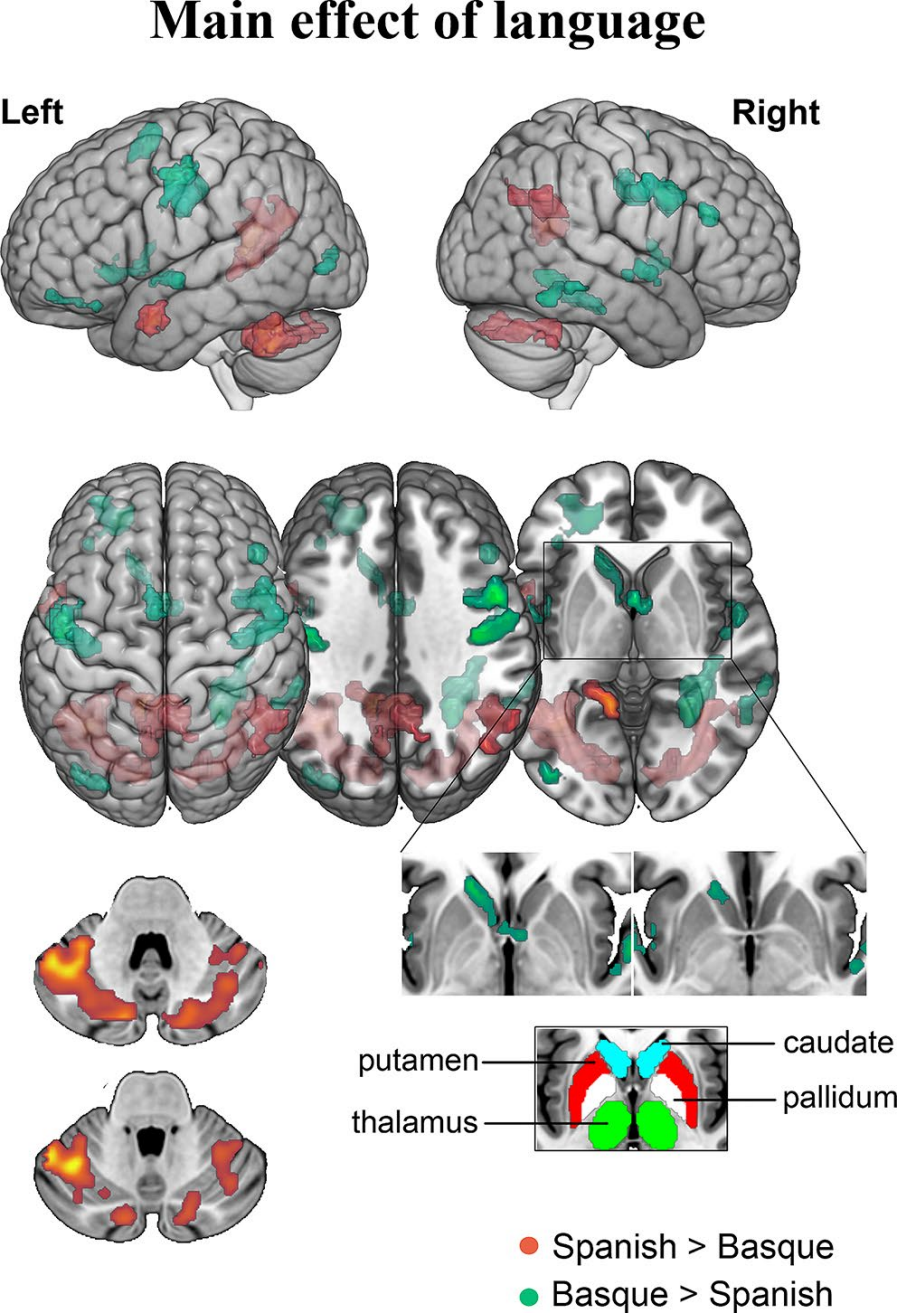
Statistical parametric map emerging from the main effect of language in healthy bilinguals. The two tails of the F-contrast are represented with different colors: Spanish > Basque in red and Basque > Spanish in green. All clusters depicted are statistically significant with a p-value corrected for multiple comparisons (FWER, *p* < 0.05)

The main effect of Language included regions with higher responses for Spanish than for Basque and regions that exhibited the opposite pattern, i.e., higher activation for Basque than for Spanish. On the one hand, significant activation increases emerged from the Spanish > Basque contrast, including the left MTG, right and left precuneus, left lingual gyrus, right angular gyrus, and the cerebellum (i.e., the right and left crus I and the right crus II). On the other hand, the contrast Basque > Spanish produced higher brain response in regions such as right and left precentral, postcentral, and STG; left IFG (pars orbitalis), left SMA, left posterior insula, left middle occipital gyrus, left caudate nucleus, right IFG (pars triangularis), right inferior temporal gyrus, and right fusiform gyrus (see Table 3 for a detailed list of regions; see Fig. 3 for a representation of response patterns). Note that these distinctions between Spanish and Basque encompass domain-general areas associated with language control and executive functions, as predicted by Tao’s et al. (2021) meta-analysis, but in addition, we also observed differences in language-specific regions.

**Table 3:** Significant activation clusters resulting from the main effect of Language

Region	Hemisp.	Coordinates			Cluster size	T-values
		x	y	z		
		Spanish > Basque				
Middle temporal gyrus	Left	-56	6	-26	142	**5.4**
Precuneus		-4	-54	14	999	**6.38**
Lingual gyrus		-10	-46	6		**6.02**
Crus I of cerebellum		-36	-56	-30	1031	**6.23**
Angular gyrus	Right	48	-54	28	321	4.49
Precuneus		4	-52	18	999	**6.5**
Crus I of cerebellum		36	-64	-34	513	4.28
Crus II of cerebellum		18	-80	-34		4.77
		**Basque > Spanish**				
Middle frontal gyrus, orbital part	Left	-22	44	-16	119	**-5.34**
Inferior frontal gyrus, pars orbitalis		-32	54	-14		-4.34
Postcentral gyrus		-50	-10	50	627	**-5.76**
Precentral gyrus		-52	0	34		-4.19
Supplementary motor area		-4	2	60	150	-4.78
Superior temporal gyrus		-54	-4	-6	94	-4.55
Posterior part of Insula		-42	4	-6		-3.74
Caudate nucleus		-16	24	-2	195	-4.84
Middle occipital gyrus		-42	-82	0	94	-4.08
Inferior frontal gyrus, pars triangularis	Right	46	28	26	72	**-5.17**
Precentral gyrus		50	4	34	640	**-5.59**
Postcentral gyrus		38	-16	38		-5.13
Fusiform gyrus		28	-50	-8	366	**-6.19**
Inferior temporal gyrus		56	-54	-18	116	-4.54
Superior temporal gyrus		52	-2	-4	81	-4.56

Only those clusters with a significant effect after FWER correction are included in the table. x,y,z (milimeters) = Coordinates in MNI space of local maxima. Cluster size = Number of voxels significantly activated inside the cluster belonging to each local maximum. T values reported in bold were also significant after FWE correction at the peak level

#### Laterality effects

A repeated measures ANOVA yielded a significant effect of language [*F*_(1,39)_ = 10.53, *p* = 0.001]. Specifically, this effect showed that Basque production additionally recruited right contralateral regions to the language-dominant hemisphere. Also, a marginally significant interaction between language and ROI was found [*F*_(1,53)_ = 71.385, *p* = 0.047].

#### Machine learning results

**Fig. 4:**
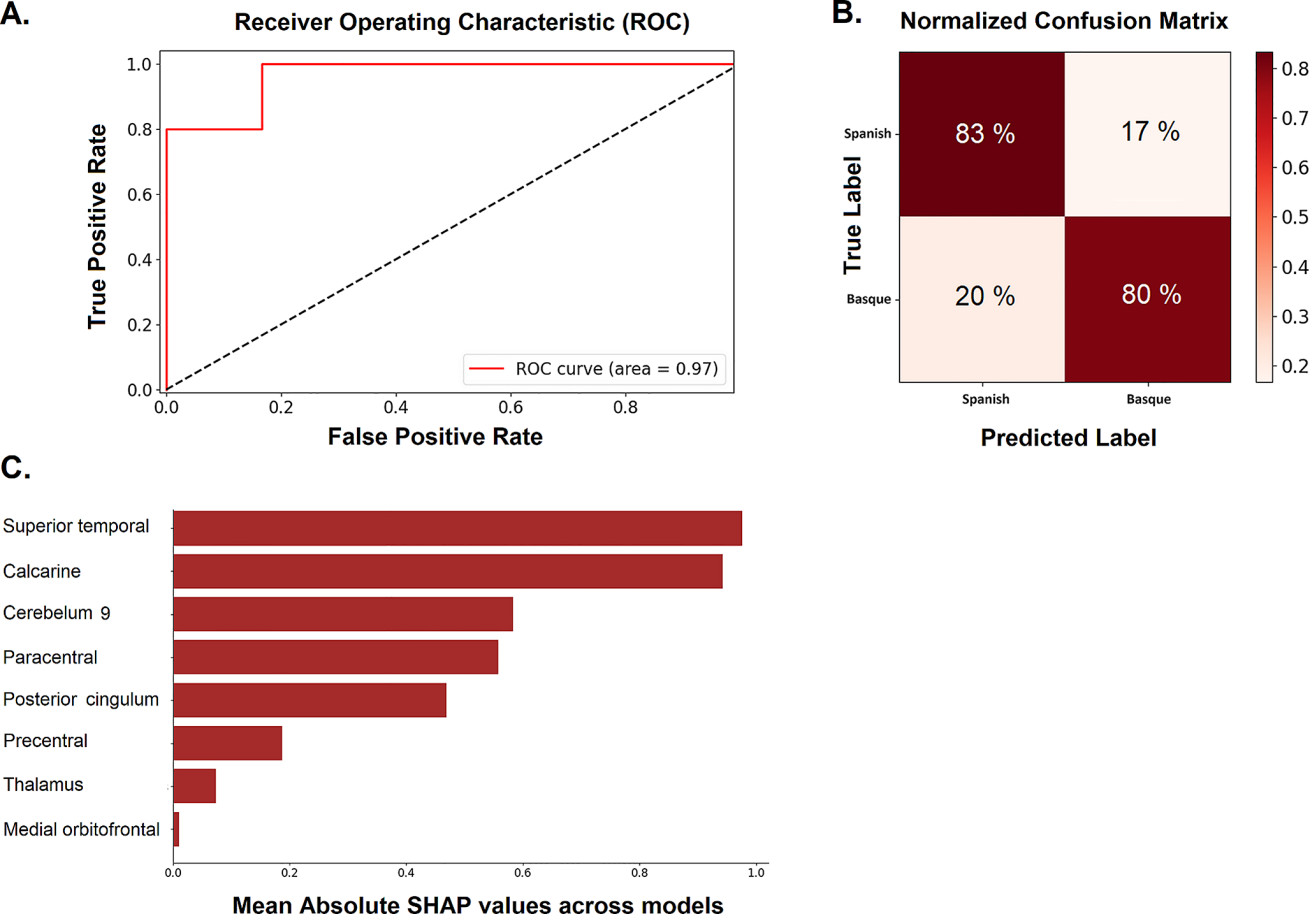
Binary classification (Spanish vs. Basque) machine learning results. (**A**) Mean area under receiver operating characteristic (ROC) curve (AUC) and (**B**) confusion matrix across models. (**C**) List of the most predictive features (in order of importance), using absolute SHAP values

The machine learning classifier distinguishing between Spanish and Basque LI obtained from BOLD activity during a speech production task, yielded an AUC of 0.80 (± 0.18), an accuracy of 74% (± 17%), a precision of 76% (± 20%), a recall of 72% (± 19%), and an F1-score of 0.73 (± 0.18). Estimates of feature importance using SHAP values highlighted that the superior temporal, calcarine, cerebellum, paracentral lobule, posterior cingulum, precentral, thalamus, and orbitofrontal cortex were the most predictive features driving classification between Spanish and Basque (see Fig. 4).

### Characterizing bilingual language production in low-grade glioma patients

#### Behavioral performance

We conducted *t*-tests on accuracy and RTs for bilingual patients’ data. Similarly to healthy controls, the results revealed no significant language effects in either accuracy (Spanish: mean = 98.72%, SD = 1.47; Basque: mean = 97.83%, SD = 2.34; t_(19)_ = 1.34, *p* = 0.26, *n*^*2*^ = 0.05) or RTs (Spanish: mean = 988.09 ms, SD = 147.29; Basque: mean = 1013.86 ms, SD = 174.64; *t*_(19)_ = 0.16, *p* = 0.69, *n*^*2*^ = 0.007).

#### Activation-based cross-language effects

**Fig. 5:**
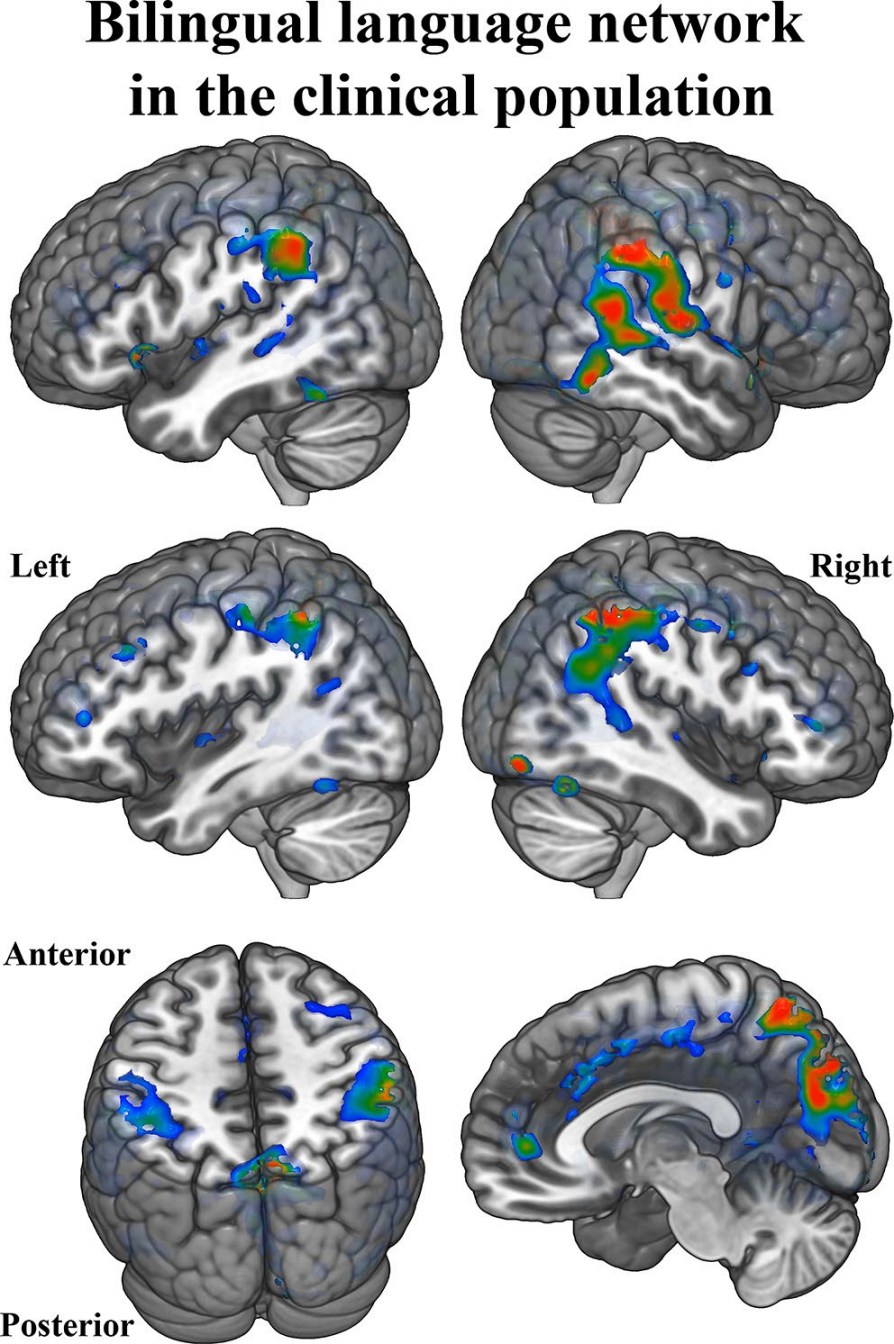
Network supporting language production in the clinical cohort. The statistical parametric map created from the contrast between all conditions versus the null distribution is projected on the MNI single-subject T1 image using the glass brain configuration parameters of MRIcroGL (https://www.nitrc.org/projects/mricrogl)

The findings from the production task in the clinical population exhibited a neural activation pattern that spanned both hemispheres. As expected, the All vs. Null contrast, to some extent, aligns with previous observations in healthy individuals. This pattern of activation encompassed several crucial areas associated with language production (see Fig. 5) including the pars orbitalis within the IFG, the SMA, the STG, and MTG, as well as the supramarginal and angular gyri. Moreover, in addition to the classical language hubs, patients exhibited a bilateral fronto-parietal network that was absent in the healthy control group. This network spatially aligns with the default mode network (DMN) and includes regions such as the precuneus, posterior cingulate, orbitofrontal/rectus, and superior parietal gyrus.

**Fig. 6:**
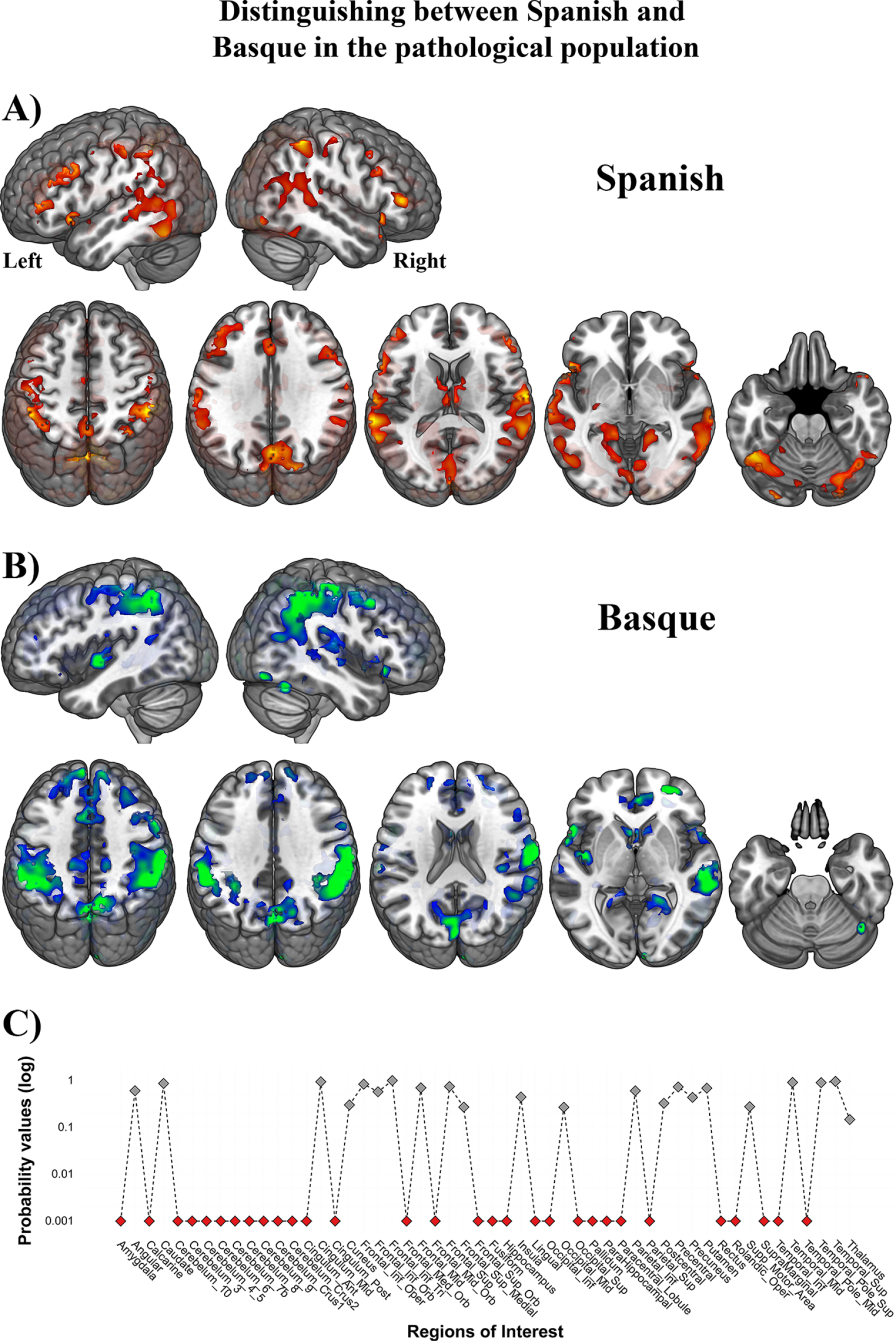
Statistical parametric map emerging from the L1 and L2 in the clinical cohort. The two contrasts are represented with different colors: **A**) Spanish (dominant language) in red and **B**) Basque (non-dominant language) in green. (**C**) Results from the permutation analysis conducted on the clinical sample to compare Spanish and Basque. The choice of this methodological approach within the clinical population is motivated by the non-normal distribution of the data, as indicated by the Shapiro-Wilk test (*p* < 0.05). The selection of global and marginal hypotheses involved combining permutation t-statistics related to univariate hypotheses through 10,000 replicates. For each repetition, maximum t-statistics were computed, forming the basis for estimating the empirical null distribution. Subsequently, p-values for the t-max statistics derived from the empirical null distribution were calculated. ROIs where the null hypothesis was rejected are denoted by red dots, with stringent control for type I error (*p* < 0.05)

When contrasting Spanish and Basque in the patient group, we uncovered noteworthy differences. Notably, for Spanish, patients engaged areas, such as the pallidum, putamen, and thalamus, which were not active for Spanish in the healthy controls. Similarly, differences were observed in the case of Basque, where patients relied on the DMN to effectively perform the task in their non-native language (see Fig. 6A and B for more details). These findings suggest the existence of distinct compensatory mechanisms operating for each language.

#### Laterality effects

**Fig. 7:**
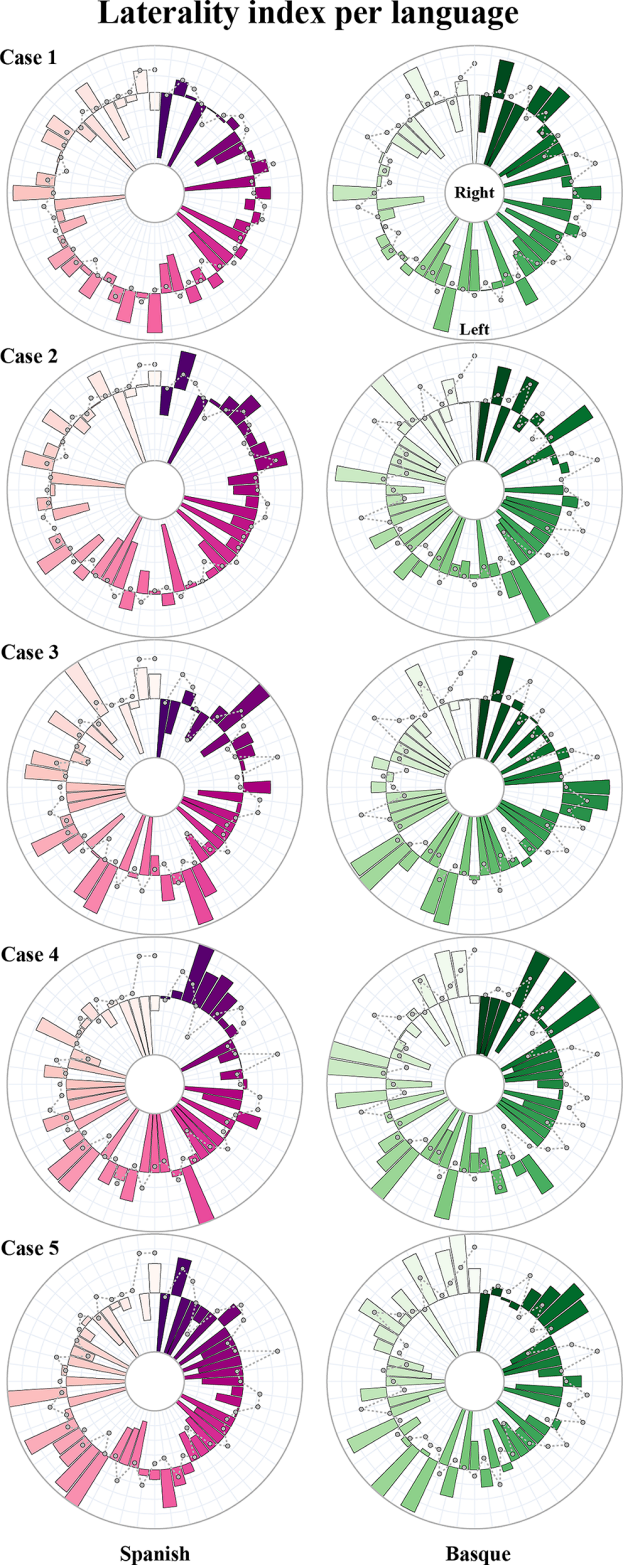
Circular charts representing laterality changes per ROI for Spanish and Basque. The middle circle represents zero laterality; bars extending from the middle circle towards the outer circle indicate increasing left laterality, while bars extending from the middle circle towards the inner circle indicate increasing right laterality

In contrast to the outcomes observed in healthy individuals, a repeated measures ANOVA using the lateralization indices in the clinical population yielded no language effect [*F*_(1,19)_ = 1.53, *p* = 0.09] and no significant interaction between language and ROI [*F*_(1,53)_ = 0.780, *p* = 0.660]. Upon delving into the individual clinical data, it was evident that the lateralization indices by region spanned the entire spectrum of possible values, ranging from − 1 (indicating full left lateralization) to 1 (indicating full right lateralization). This inter-individual variability is visually represented in Fig. 7, where each circular chart corresponds to a different patient. However, despite this inter-individual variability, discernible language-related changes were evident across ROIs. To better characterize these ROI-based patterns within the clinical population, we employed a permutation analysis to determine which regions consistently exhibited language effects across different individuals (refer to Fig. 7). Notably, this analysis unveiled that regions like the cerebellum, fusiform gyrus, orbitofrontal cortex, superior parietal, and supramarginal gyrus exhibited differential activation in response to Spanish and Basque.

## Discussion

The aims of the current study were twofold: (i) to characterize the functional organization that enables the coexistence of two languages in the bilingual brain and (ii) to scrutinize the impact of neuroplastic mechanisms induced by brain tumors on this complex organization. A notable aspect of our study lies in the integration of univariate and multivariate methodologies, aiming to discern unique neural patterns for both languages in simultaneous bilinguals. Unlike previous research, we examined both languages within the same individuals but in separate sessions, thereby minimizing potential mechanisms of competition or inhibition between languages (see Bruin et al. 2021; DeLuca et al. 2020; Pliatsikas 2020 for reviews on this topic). Furthermore, the use of low-grade gliomas as a pathological model of neuroplasticity offered us a unique opportunity to delineate the adaptability of the network associated with language production emphasizing aspects related to the bilingual brain.

Specifically, our findings in healthy bilinguals reveal that, despite the existence of a common neural substrate supporting both languages, Spanish and Basque recruit different brain regions during speech production. While the dominant language (Spanish) engaged a more left-lateralized network, the non-dominant (Basque) relied on the recruitment of a bilateral basal ganglia-thalamo-cortical circuit. Notably, based on language lateralization patterns, we were able to robustly decode (AUC: 0.80 ± 0.18) the language being used. Conversely, bilingual patients exhibited bilateral activation patterns for both languages, indicating that pre-surgical neuroplastic changes required to preserve bilingual speech production involve compensatory engagement of contralateral networks, irrespective of the language being used. Nonetheless, while contralateral compensation mechanisms impact both languages, the neural networks processing each language differed. Collectively, these findings suggest that language production is rooted in a remarkably adaptable system, capable of adjusting to diverse contexts and demands (Chinichian et al. 2023; Nieberlein et al. 2023; Roger et al. 2022; Yuan et al. 2023). This adaptability accounts for the reliance on a specialized left lateralized perisylvian system for production in the dominant language, whereas speaking in the non-dominant one depends on a broader bilateral anatomical substrate. This flexibility also explains the intriguing phenomenon of functional compensatory reorganization observed in individuals with brain tumors. Each result is discussed in detail below.

### Investigating the bilingual language network in healthy individuals

Our results concerning the healthy population show that bilingual sentence production, despite its left-lateralized nature, engages regions from both hemispheres. These regions extend beyond the well-known perisylvian areas, revealing a flexible system that requires the interaction of multiple neural networks. Consistent with previous models, this system involves frontal, temporal, and parietal regions, as well as the cerebellum, the hippocampus, and basal ganglia nuclei, such as the thalamus, caudate, and pallidum (Hickok and Poeppel 2007; Piai and Zheng 2019; Poeppel 2014b; Yuan et al. 2022). Strikingly, our findings also unveil that the observed pattern undergoes adaptations depending on the language being used, showcasing a predominantly left-lateralized pattern for the dominant-language consistent with previous studies (Groen et al. 2012; Gurunandan et al. 2020; Jia et al. 2022; Parker et al. 2022) and a more bilateral pattern for the non-dominant one. Hence, dual-language experience plays a pivotal role in shaping the dynamics of the language network: to effectively produce speech in a non-dominant language, the brain must recruit additional cortical and subcortical areas that remain inactive when using the dominant language.

Notably, our machine-learning approach achieved a very good performance (AUC: 0.80 ± 0.18) in distinguishing between Spanish and Basque (Davatzikos 2019; Singh et al. 2022), with eight brain regions providing maximal discrimination between them (see Booth et al. 2007 for a review). These regions align well with those identified by the univariate analysis, spanning cortical (e.g., superior temporal cortex, calcarine, orbitofrontal cortex, and posterior cingulum) and subcortical structures (cerebellum and thalamus). In line with our cortical findings, Xu et al. (2017) conducted a multivoxel pattern whole-brain analysis in English-Chinese bilinguals and successfully differentiated subsets of voxels that overlap for Spanish and Basque from those subsets with cross-language classificatory capacity, including occipito-temporal and orbitofrontal structures (also see DeLuca et al. 2019). When considering subcortical findings, as far as the basal ganglia nuclei are concerned, their role in language processing was initially proposed by Ullman (2001, 2004) who incorporated these areas into the declarative/procedural model of the acquisition of the L2 lexicon and grammar (Ullman 2001, 2004). In this model, lexical access and grammar comprehension depend on two distinct memory systems: associative memory which involves the medial temporal lobe, and procedural memory which is supported by a basal ganglia-thalamo-cortical loop specialized in processing hierarchical grammatical structures. More recently, the authors have extended this model by suggesting that this circuit serves as the computational system for language coding, operating within a structure of working memory networks (Morgan-Short and Ullman 2022; Ullman 2020). Even when this model is primarily based on bilinguals who have acquired the two languages sequentially, our results support this interpretation: a basal ganglia-thalamo-cortical circuit is recruited for the non-dominant language with greater amplitude than for the dominant one. Within this circuit, the thalamus seems to play a role as the regulator of interhemispheric information flow and cortico-subcortical connectivity (DeLuca et al. 2019, 2020; Morgan-Short et al. 2022; Morgan-Short and Ullman 2022; Pliatsikas et al. 2017; Ullman 2020). It is intriguing that in simultaneous bilinguals, even when controlling for AoA, proficiency, and exposure—factors known to influence neural distance between languages—we observe results similar to those seen in sequential bilinguals. What, then, determines the neural dominance of one language over the other in simultaneous bilinguals? Considering language dominance as a dynamic factor that varies with context (e.g., the interlocutor’s identity or the location) could explain the neural cost associated, in this particular case, with the processing of Basque. This is particularly relevant for highly proficient simultaneous bilinguals who regularly alternate between languages based on the context in a bilingual environment. Interestingly, this cross-language variability also aligns with the findings of the SenSAAS functional language atlas outlined by Labache et al. (2019). Specifically, these changes are concentrated within what Labache et al. termed the “memory” and “visual processing cores,” as delineated through cluster analysis. These cores encompass regions exhibiting considerable variability among individuals, suggesting that within the networks that support linguistic processing, cores with greater flexibility could be distinguished. In line with these proposals, Korenar et al. (2021) demonstrated how bilingual experiences induce dynamic structural changes in the caudate and thalamus (see also Korenar et al. 2023). These areas, which have been extensively studied in both healthy and clinical populations, are known to play a crucial role in language control processes (see also DeLuca et al. 2019, 2020; Pliatsikas et al. 2017). For instance, thalamic lesions have been associated with lexical-semantic impairments (Klostermann et al. 2022), while damage to the caudate has been linked to disruptions in language control (Aglioti et al. 1996; Lee et al. 2016). In studies involving healthy individuals, the caudate has been reported to play a central role in language control tasks such as translation (Mouthon et al. 2020), language selection (Branzi et al. 2016), and language switching (Abutalebi et al. 2013).

### Investigating neuroplasticity in bilingual patients with low-grade gliomas

In the context of neuroplasticity in bilingual patients with low-grade gliomas, the current study achieved several significant findings. First, it successfully validated MULTIMAP (Gisbert-Muñoz et al. 2021) as a tool for functionally delineating the network supporting bilingual speech production. Second, it demonstrated the presence of neuroplasticity mechanisms associated with tumor growth, which involved the recruitment of healthy ipsilesional and contralateral homologous areas. Lastly, the study revealed that these neuroplastic mechanisms had distinct effects on the patients’ dominant and non-dominant languages. The analysis of functional lateralization indicated that both languages exhibited greater recruitment of regions in the right hemisphere compared to the healthy control group. These distinct patterns of reorganization suggest that different compensatory networks might come into play to preserve bilingual functions. Consistently, evidence from direct cortical stimulation during awake craniotomies on bilinguals (Giussani et al. 2007; Gomez-Andres et al. 2022; Lubrano et al. 2014; Sierpowska et al. 2018) has demonstrated that although there are common areas for both languages, there are also cortical territories that preferentially respond to one language or another (Paradis 2008). In support of these results, previous studies on bilingual patients with brain tumors have reported post-operative language impairment in only one language (Fabbro 2001).

Integrating findings from both healthy and clinical populations underscores the adaptive nature of the language production network. This network possesses self-adaptive mechanisms that enable it to effectively respond to endogenous events, such as developing oncological lesions, and exogenous events, such as switching between languages depending on the context. In the case of healthy individuals, the associated increases in cognitive demands are mitigated through the engagement of a cortico-subcortical network. This network comprises structures like the thalamus, caudate, and cerebellum, along with the recruitment of contralateral counterparts from language-critical regions such as the IFG, SMA, and MTG. An intriguing observation arises when examining patients with gliomas, as the activation map for their dominant language already involves these supportive structures. The presence of glioma seems then to impose an additional burden on the system, requiring compensatory recruitment of this cortico-subcortical network. However, when delving into the analysis of the non-dominant language in the clinical population, a different pattern emerged. Here, the recruitment shifts towards domain-general structures that lack a direct connection to language and originate from the DMN. It appears that the cortico-subcortical network, as previously engaged, is no longer sufficient to support speech production in the context of a non-dominant language. This observation marks a pivotal departure in the approach to surgical interventions for this class of brain injury. In pursuit of favorable post-surgical outcomes, consideration of the system’s functional reorganization prior to diagnosis becomes imperative. These alterations might extend beyond the boundaries of the affected network. In line with this proposal, a recent comprehensive review on neuroplasticity in gliomas has suggested that compensatory reorganization following such lesions could potentially hinge on the engagement of general amodal networks not exclusively tied to specific cognitive functions (Duffau 2017, 2021; Nieberlein et al. 2023). Further research should prioritize the investigation of the short, medium, and long-term consequences associated with the sustained activation of the DMN in this context. This exploration will yield valuable insights into the implications of such recruitment strategies on recovery processes.

### Implications for surgical practice

Language production is key to achieving the professional and personal reinsertion of glioma patients. However, even though advances in surgical techniques and intraoperative management have resulted in an exponential decrease in the appearance of serious cognitive sequelae (Duffau 2017), ~ 90% of glioma patients who undergo surgery are affected by some cognitive symptomatology that hinders their professional reintegration and the recovery of their personal life (Loon et al. 2015; Tucha et al. 2000). Previous studies have reported language and memory impairment as the two most frequently occurring cognitive deficits, followed by deficits in executive functions and visuospatial abilities (Zucchella et al. 2013).

The methodology presented in this study offers a highly valuable tool for neurosurgery departments. It underscores the importance of conducting a comprehensive multilingual pre-surgical assessment for bilingual patients, thereby facilitating personalized intervention planning. This approach enables a more comprehensive identification of functionally active language areas, which may otherwise be overlooked in single-language mapping. It also prompts a reevaluation of intraoperative language mapping protocols for awake brain surgery, uncovering new indications for areas that were not traditionally deemed eloquent for language. The availability of tools like MULTIMAP (Gisbert-Muñoz et al. 2021), which not only evaluates the pre-surgical lateralization of both languages but also assesses neuroplastic changes resulting from pathological conditions, is crucial for devising personalized intervention strategies. Furthermore, the activation of regions contralateral to the tumor has previously been shown to be a reliable predictor of post-surgical recovery (Oda et al. 2018). Therefore, preserving areas that have undergone neuroplastic changes before surgery plays a pivotal role in achieving favorable postoperative results.

### Future directions

By taking advantage of simultaneous bilinguals, novel theoretical questions in the field of brain cognition and neuroplasticity can be addressed: Are individual differences in linguistic profiles causally linked to variations in network flexibility? Does network flexibility represent an adaptive advantage applicable to both learning and pathological situations? What are the short-, medium- and long-term consequences of sustained DMN activation as a compensatory mechanism? By answering these questions in future studies, it will be possible to improve clinical interventions promoting longer life expectancy and better quality of life for patients. From a methodological perspective, these analyses could also benefit from using more specialized functional atlases tailored to language networks (Labache et al. 2019; Lipkin et al. 2022). Such atlases offer enhanced precision by estimating the probability that specific locations within a common space fall within the language network. By leveraging these atlases, future studies can further evaluate the replicability of our findings.

## Conclusions

In this study, we have demonstrated that despite the similarities in the bilingual participants’ performance across languages, their dual-language experience has indeed caused an imprint at the neuro-anatomical level. This imprint not only impacts domain-general areas associated with executive functions but is also reflected in changes in the functional dynamics of language-specific regions. Our findings suggest that, under typical conditions, processing a non-dominant language requires the involvement of a basal ganglia-thalamo-cortical circuit, which is not necessary for producing the dominant one. However, the analysis of patients with brain tumors reveals a remarkable flexibility within the production network that impacts both languages, albeit with some language-specific distinctions. Taken together, this evidence underscores the existence of a flexible bilingual speech production system, which extends beyond left perisylvian areas. This system includes self-adaptive mechanisms that ensure effective responses in both health and disease. These results have significant implications for the preoperative evaluation and subsequent treatment of bilingual patients with low-grade gliomas. By highlighting the importance of considering individual linguistic phenotypes in the design of personalized interventions, our study will directly impact the care and management of these patients. The ability to identify the specific brain regions crucial for bilingual language processing opens new possibilities for targeted and effective therapeutic approaches, ultimately enhancing patient outcomes.

## Data Availability

No datasets were generated or analysed during the current study.

## Footnotes

1 https://www.euskadi.eus/contenidos/informacion/eas_ikerketak/es_def/adjuntos/VII-ENCUESTA-SOCIOLINGUISTICA_resumen-.pdf.
